# Altered Fetal Head Growth in Preeclampsia: A Retrospective Cohort Proof-Of-Concept Study

**DOI:** 10.3389/fped.2015.00083

**Published:** 2015-10-07

**Authors:** David P. Eviston, Anna Minasyan, Kristy P. Mann, Michael J. Peek, Ralph K. Heinrich Nanan

**Affiliations:** ^1^Discipline of Obstetrics, Gynaecology and Neonatology, Charles Perkins Centre Nepean, Sydney Medical School Nepean, The University of Sydney, Penrith, NSW, Australia; ^2^Discipline of Paediatrics and Child Health, Charles Perkins Centre Nepean, Sydney Medical School Nepean, The University of Sydney, Penrith, NSW, Australia; ^3^NHMRC Clinical Trials Centre, The University of Sydney, Camperdown, NSW, Australia

**Keywords:** fetus, cephalometry, preeclampsia, head, neurotrophins, growth, *in utero*, ultrasound imaging

## Abstract

**Background:**

Preeclampsia is associated with fetal growth restriction and low birth weights. Neurotrophins, which mediate neuronal growth and development, are also increased in the placenta and cord blood in preeclampsia. Hence, the aim of this study was to determine whether fetal head growth is altered in preeclampsia, adjusting for growth restriction and other confounding variables.

**Methods:**

This research included a retrospective cohort study, looking at fetal head circumference at birth, plus a case–control study examining fetal head circumference at mid-gestation. The head circumference at birth analysis consisted of 14,607 pregnancies (preeclampsia = 382, control = 14,225), delivered between July 2006 and June 2012 at Nepean Hospital, Australia. Head circumference at birth, in addition to other maternal and fetal variables, was sourced from the Nepean Obstetric Database. The head circumference at mid-gestation study consisted of 756 pregnancies (preeclampsia = 248, control = 508), delivered within the same data collection period at Nepean Hospital. Head circumference at mid-gestation was retrieved from an earlier ultrasound scan. Exclusion criteria included >1 fetus, illegal drug use, alcohol consumption, and chronic or gestational hypertension. Generalized linear models were used to analyze fetal head circumference in preeclampsia versus controls, adjusting for confounding variables.

**Results:**

Head circumference increased at a greater rate in preeclampsia versus controls, adjusted for gestation, fetal gender, birth weight and length, smoking, maternal BMI, and growth restriction. At mid-gestation, there was no difference in head circumference between preeclampsia and controls.

**Conclusion:**

For the first time, this research has suggested increased fetal head growth in preeclampsia, adjusted for confounders. This finding may be explained by altered fetal exposure to neurotrophins in preeclampsia. The long-term neurodevelopmental consequences of preeclampsia remain unclear.

## Introduction

Preeclampsia is a leading cause of fetomaternal morbidity and mortality. The exact etiology of preeclampsia remains unsolved, though placental dysfunction is central. Specifically, impaired implantation results in an abnormally shallow placenta, with potential for vascular insufficiency and subsequent fetal growth disturbance (1, 2). Additional to low birth weights, preeclampsia is associated with asymmetric growth restriction, whereby fetal brain growth is maintained at the expense of less critical organs (3, 4). Accordingly, fetal head growth might be altered in preeclampsia; however, this has not been well established (5).

More than nutrient supply, brain growth is determined by neurotrophins, including brain-derived neurotrophic factor (BDNF). As key mediators of neuronal growth, disorders associated with alterations in BDNF are linked to changes in brain volume (6, 7). In preeclampsia, placental and cord blood BDNF levels are also increased (8, 9), with possible consequences for fetal neurodevelopment.

Head circumference closely relates to brain volume and is routinely performed on all newborns (10). The aim of this study was to introduce the concept of altered fetal head growth in preeclampsia. It was hypothesized that fetal head growth would be increased in preeclampsia, adjusting for growth restriction and other confounding variables.

## Materials and Methods

This research included a retrospective cohort study examining fetal head circumference at birth plus a case–control study examining head circumference at mid-gestation. Singleton pregnancies delivered at Nepean Hospital, Australia, between July 1, 2006, and June 30, 2012, were considered eligible. Each included pregnancy had a single normal fetus. Exclusion criteria were chronic or gestational hypertension, alcohol consumption, and illegal drug use. Pregnancy information was sourced from the Nepean Obstetric Database, the information in which is updated and maintained during and after pregnancy and contributes to state-wide data collection.

The Ethics Committee of the Sydney West Area Health Service approved this study according to the Declaration of Helsinki.

Preeclampsia was defined as *de novo* hypertension (systolic BP ≥ 140 mmHg and/or diastolic BP ≥ 90 mmHg) and proteinuria (≥0.3 g/24 h or spot urine protein/creatinine ratio ≥ 30 mg/mmol) after 20 weeks’ gestation, in accordance with recommendations proposed by the International Society for the Study of Hypertension in Pregnancy (11).

**Figure 1:**
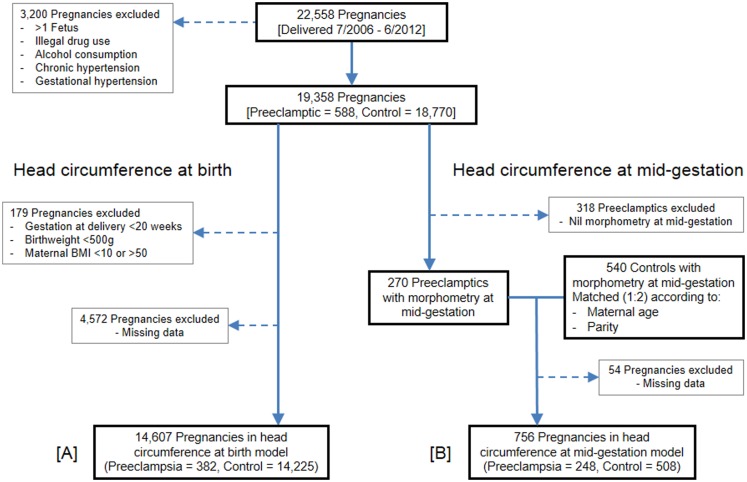
**Flow of pregnancies for inclusion**. **(A)** Head circumference at birth population. **(B)** Head circumference at mid-gestation population.

In the head circumference at birth analysis (Figure 1A), additional exclusion criteria were applied; these being, maternal BMI <10 or >50, gestation at birth <20 weeks, and birth weight <500 g.

The head circumference at mid-gestation analysis (Figure 1B) followed the same exclusion criteria as the head circumference at birth analysis, except cutoffs for maternal BMI, gestation, and birth weight were not applied. Viewpoint software was used to retrieve fetal morphometry from mid-gestation ultrasound scans, with pregnancies lacking such data excluded. Remaining preeclamptic pregnancies were matched (1:2) with control pregnancies according to maternal age and parity. Control pregnancies were delivered within the same data collection period and followed the same exclusion criteria. For each preeclamptic case, matched controls were the first two pregnancies encountered from a random list, which also had fetal morphometry data available from a mid-gestation scan.

Comparisons of maternal and fetal characteristics were performed using GraphPad Prism 6 for Windows (Version 6.01; GraphPad Software, Inc., La Jolla, CA, USA).

Continuous maternal and fetal characteristics were compared between groups using Mann–Whitney *U* test, as the normality assumption was violated. Categorical characteristics were compared using either Fisher’s exact test or Chi-square test, depending on group size.

A multivariate generalized linear model (GLM) was used to model head circumference at birth. Given that mother could be in the database more than once (if the mother had more than one child in the study period), this correlation was accounted for in the model. The effect of preeclampsia was investigated adjusted for gestational age, fetal gender, birth weight, birth length, and fetal growth restriction, as well as the mother’s BMI and whether they smoked during their pregnancy. An interaction between preeclampsia and gestational age was also included in the model to investigate the rate at which head circumference at birth increased.

Another GLM was used to model head circumference at mid-gestation. The effect of preeclampsia was adjusted for gestational age at ultrasound, fetal gender, maternal BMI, and smoking status during pregnancy.

For all analyzes, a two-sided *p*-value <0.05 was considered statistically significant. No corrections have been made for multiple comparisons. SAS v9.3 (SAS Institute Inc., Cary, NC, USA) was used for the GLM models.

## Results

Between July 1, 2006, and June 30, 2012, 22,558 pregnancies were delivered at Nepean Hospital, 753 of which were diagnosed with preeclampsia. Following application of the exclusion criteria, 19,358 pregnancies remained eligible: 588 preeclamptics and 18,770 controls. Enforcement of maternal BMI, gestation, and birth weight cutoffs excluded a further 179 pregnancies. Of the remaining eligible pregnancies, 4,572 were excluded from the model due to missing data, mostly owing to blank data entry for fetal growth restriction and/or maternal BMI. The head circumference at birth GLM therefore consisted of 14,607 pregnancies: 382 preeclamptics and 14,225 controls.

The head circumference at mid-gestation analysis commenced with the same 588 preeclamptics which remained following application of the exclusion criteria. Of these, 318 preeclamptics were ineligible because they lacked fetal morphometry data from a mid-gestation scan. The remaining 270 preeclamptics were matched (1:2) with 540 controls, which also contained fetal morphometry data from a mid-gestation scan. Due to missing data, especially maternal BMI, 54 pregnancies were finally excluded. The GLM for head circumference at mid-gestation thus consisted of 756 pregnancies: 248 preeclamptics and 508 controls.

In the head circumference at birth population, preeclampsia was associated with an earlier gestation at delivery, increased maternal BMI, nulliparity and smoking, plus decreased birth weight, child length, and head circumference parameters (Table 1).

**Table 1:** **Maternal and fetal characteristics**.

Characteristic	Head circumference at birth population			Head circumference at mid-gestation population		
	Preeclampsia (382)	Control (14,225)	*p-*value	Preeclampsia (248)	Control (508)	*p-*value
Maternal age (yr)	28.0 (23.0–33.0)	28.0 (24.0–32.0)	0.12	28.0 (23.3–33.0)	28.0 (23.3–33.0)	0.76^a^
Maternal BMI (kg/m^2^)	28.1 (23.7–34.6)	24.5 (21.4–29.4)	<0.001	27.9 (23.8–34.8)	24.2 (21.2–29.1)	<0.001
Nulliparous	45.5%	25.5%	<0.001^c^	60.5%	59.4%	0.81^a^^,^^b^
Smoking	12.0%	20.9%	<0.001^c^	8.1%	17.3%	<0.001^b^
Male fetus	53.1%	51.4%	0.55^c^	54.0%	51.8%	0.59^b^
Fetal growth restriction	13.1%	2.4%	<0.001^c^	11.6%	1.3%	<0.001^b^
**Fetal morphometry at birth**						
Gestation at delivery (wk)	38.1 (36.0–39.3)	39.7 (38.9–40.7)	<0.001	38.1 (36.2–39.2)	39.6 (39.0–40.5)	<0.001
Birth weight (g)	3,100 (2,512–3,546)	3,465 (3,140–3,785)	<0.001	3,183 (2,556–3,609)	3,460 (3,115–3,730)	<0.001
Child length (cm); *N*	49.0 (46.0–52.0)	51.0 (49.0–53.0)	<0.001	50.0 (47.0–52.0); 240	51.0 (49.0–53.0); 502	<0.001
Head circumference (cm); *N*	34.0 (32.4–35.3)	34.5 (33.5–35.5)	<0.001	34.5 (32.8–35.5); 241	34.5 (33.5–35.5); 502	0.02
**Fetal morphometry at mid-gestation**						
Gestation at ultrasound				19.1 (18.7–19.6)	19.2 (18.9–19.6)	0.31
Head circumference (mm)				161.5 (156.4–168.4)	162.3 (156.8–169.0)	0.37
Biparietal diameter (mm)				43.6 (42.0–45.8)	43.9 (41.8–45.7)	0.89
Abdominal circumference (mm)				141.6 (135.5–148.4)	143.2 (135.8–150.3)	0.22
Femur length (mm)				29.5 (28.0–31.5)	29.9 (28.3–31.5)	0.26

*^a^Matched variables*.

*^b^Fisher’s exact test*.

*^c^Chi-square test; continuous data analyzed using Mann–Whitney *U* test and expressed as median (Q1–Q3)*.

At mid-gestation, the preeclampsia group also demonstrated increased maternal BMI and smoking; however, fetal morphometry was comparable.

**Figure 2:**
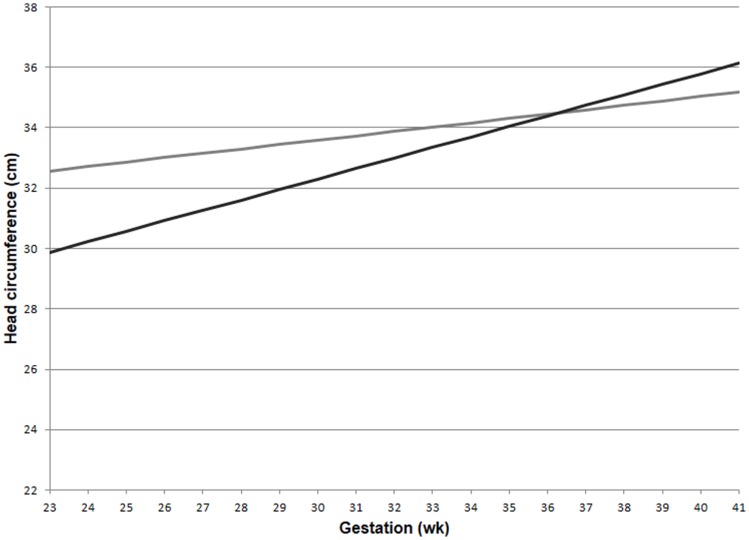
**Adjusted means for fetal head circumference at birth for preeclampics and controls over gestational ages**. Head circumference increased faster in preeclampsia versus controls (interaction *p*-value < 0.001). This comparison assumed a male baby of average birth weight and length, with growth restriction, average maternal BMI, and a non-smoking mother. Preeclampsia (–); control (–).

The GLM for head circumference at birth, which included an interaction between preeclampsia and gestational age, revealed a significantly increased slope in the preeclampsia group versus controls over the gestational ages modeled (Figure 2; interaction *p*-value <0.001). The lines are shown to cross at 36.3 weeks gestation. Before this time, a delivered preeclamptic fetus is estimated to have a smaller fetal head circumference than a control fetus (adjusting for confounders), and the difference between the two fetuses becomes smaller as gestational age increases. Conversely, a preeclamptic fetus born after 36.3 weeks gestation is estimated to have a larger head circumference than a control fetus, and this difference increases with increasing gestational age. Table 2 illustrates a comparison of head circumference adjusted means, for that average baby in Figure 2, at representative gestations.

**Table 2:** **Comparison of adjusted mean fetal head circumference**.

Gestation (wk)	Head circumference (cm)	
	Preeclampsia	Control
24	30.2	32.7
28	31.6	33.3
32	33.0	33.9
36	34.4	34.4
40	35.8	35.0

*Adjusted for fetal gender, birth weight, birth length, fetal growth restriction, maternal BMI, and smoking*.

Head circumference at mid-gestation was comparable between preeclampsia and control groups, adjusting for gestation at ultrasound, maternal BMI, smoking, and fetal gender [estimated mean difference (95% CI) = 0.003 higher in preeclampics (−0.08 to −0.09); *p* = 0.94].

## Discussion

For the first time, this study has suggested fetal head circumference to increase at a greater rate in preeclampsia, adjusting for growth restriction and other confounding variables. At mid-gestation, fetal head circumference was comparable between preeclampsia and control groups.

A major strength of this study is large sample size; however, numerical errors made during data entry became a concern. To address this issue, cutoff criteria for maternal BMI, gestation, and birth weight were applied, the aim being to eliminate incorrect data. The application of cutoffs is unlikely to have introduced significant bias, however, since broad limits were used and relatively few pregnancies were excluded. Indeed, most excluded pregnancies were due to missing data, especially maternal BMI. Since the head circumference at birth model consisted of one measurement for each fetus, caution should be observed in interpreting head circumference changes as changes in velocity. Nonetheless, repeated measures are not feasible on a large sample size and despite its limitations, cross-sectional birth data are generally accepted as the best method of generating growth curves (12). Finally, extension should not be made outside the gestational ages modeled here in this article.

The purpose of this research was to introduce the novel concept of altered fetal head growth in preeclampsia. As mid-gestation head circumference measures were comparable in this study, and birth head circumference measures indicated an increased head growth in preeclampsia, altered neurodevelopment might be a late event. However, the gestation at birth ranges in this study was relatively narrow and different between groups. Hence, further research is required to establish fetal head growth in preeclampsia versus healthy pregnancy, which might include serial measures of head circumference *in utero*.

Similar to previous reports, male gender and increased maternal BMI were associated with larger head circumference at birth in this study (12). Birth weight was also smaller in preeclamptic fetuses, again consistent with earlier findings (5, 13). However, there are few published studies on head growth in preeclampsia and none adjusted for confounding variables (14). Comparison of our results with previous findings is therefore restricted.

Preeclampsia is associated with altered fetal growth patterns. In particular, it is linked to small for gestational age (SGA) fetuses; a finding frequently explained by decreased placental perfusion (2, 5, 13). In chronic placental insufficiency, head growth may be spared at the expense of less critical tissues, resulting in asymmetric growth restriction. However, this study observed relatively increased head growth in preeclampsia, adjusting for growth restriction. An alternative mechanism may therefore explain our findings.

Altered head growth in preeclampsia may be explained by increased fetal exposure to neurotrophins, including BDNF. An essential regulator of normal neurodevelopment, disorders linked to alterations in BDNF demonstrate changes in brain growth (6, 7, 15). In term preeclampsia, elevated BDNF is also observed in both the placenta and the cord blood (8, 9). Accordingly, increased fetal exposure to BDNF at term may explain increased head growth in preeclampsia, although further studies are needed.

In conclusion, for the first time, this study has suggested fetal head growth to be increased in preeclampsia, adjusted for confounding variables. Further investigation is required, and if these findings are confirmed, the long-term neurodevelopmental consequences of preeclampsia will be the subject of future research efforts.

## Author Contributions

DE contributed to data collection, manuscript preparation, statistics, and final approval. AM contributed to study design, revision of manuscript, and final approval. KM contributed to statistics, review of manuscript, and final approval. MP contributed to study design, revision of manuscript, and final approval. RN contributed to study design, revision of manuscript, and final approval.

## Conflict of Interest Statement

The authors declare that the research was conducted in the absence of any commercial or financial relationships that could be construed as a potential conflict of interest.
